# Hydration Resistance of CaO Material Prepared by Ca(OH)_2_ Calcination with Chelating Compound

**DOI:** 10.3390/ma12142325

**Published:** 2019-07-22

**Authors:** Jinhu Wang, Yaowu Wei, Nan Li, Junfeng Chen

**Affiliations:** The State Key Laboratory of Refractories and Metallurgy, Wuhan University of Science and Technology, Wuhan 430081, China

**Keywords:** CaO material, hydration resistance, chelating compound, surface pretreatment

## Abstract

The hydration resistance of CaO materials prepared by Ca(OH)_2_ calcination with chelating compounds are investigated in this paper. The crystalline phases and microstructure characteristics of sintered specimens were studied by X-ray diffraction (XRD), X-ray photoelectron spectroscopy (XPS), scanning electron microscopy and energy dispersive spectrometer (SEM, EDS). The bulk density, apparent porosity, and hydration resistance of samples were also tested. The results showed that chelating compounds improved the hydration resistance of the treated CaO specimens significantly. The surface-pretreated specimens showed an increase in bulk density and a decrease in apparent porosity after heating. The surface pretreatment of the Ti chelating compound promoted the solid phase sintering and grain growth of CaO specimens, which increased the density of the heated CaO sample. The Al chelating compound promoted the liquid-phase sintering of CaO specimens, which led to the grain growth and increased density of the sample. CaO grains were bonded by the formed tricalcium aluminate (C_3_A) and the apparent porosity of the sample was reduced, reducing the contact area of CaO with water vapor. The Al chelating compound was more effective in improving the hydration resistance of the CaO material in the situation of this study.

## 1. Introduction

CaO refractories have excellent properties such as high stability, low vapor pressure at high temperature, good slag resistance, high refractoriness, and excellent action in purifying molten steel [1,2,3]. It does not pollute the molten steel but absorbs non-metallic inclusions such as S, P, Al_2_O_3_ in the molten steel [4,5,6]. In addition, it is one of the best materials for preparing crucibles, filters, and nozzles used for metallurgical purposes. However, CaO’s easy hydration characteristic is a limiting factor in many applications, as well as in storage and preparation. The research on improving the hydration resistance of CaO refractories has been uninterrupted in the past thirty years, and there are two main methods to enhance the hydration resistance of CaO materials. The first method consists of introducing additives by promoting solid-phase sintering (e.g., ZrO_2_ [7,8,9], TiO_2_ [10], rare earth oxides La_2_O_3_ [11] and CeO_2_ [4,12]) or liquid-phase sintering (e.g., Al_2_O_3_ [13], Fe_2_O_3_ [14,15], CuO [16], V_2_O_5_ [17], Cr_2_O_3_ [18], etc.) which will respectively produce CaO materials with a denser structure, or form a low-melting-point phase to coat the surface of the CaO grain. Kahrizsangi et al. reported that the addition of nano-TiO_2_ to a magnesite-dolomite refractory matrix helped in the densification process by solid-state sintering and increased the hydration resistance [10]. Wei et al. studied the effects of Zr(OH)_4_ and Al(OH)_3_ on the hydration resistance of CaO granules. The results showed that the hydration resistance of CaO granules was improved significantly [19]. Those methods reduced the contact area of CaO with water vapor and improved the hydration resistance of CaO materials. The formation of vitreous phases was conducive to a better resistance to hydration [20]. The second method is surface treatments such as carbonation, phosphate treatment, siloxane solutions, or anhydrous organics [21,22,23]. Chen et al. investigated the effect of carbonation on the hydration resistance of CaO materials and established that carbonation treatment effectively improved the hydration resistance of CaO materials [24].

A new idea is introduced in this paper in order to prolong the shelf life of CaO materials. Ca(OH)_2_ was selected as the raw material for the preparation of CaO sample in this study. The shaped Ca(OH)_2_ sample was pretreated with chelating compound first and then heated at high temperature. The chelating compound formed a good coating by physical and chemical reactions with Ca(OH)_2_. The chelating compounds used in this paper decomposed between 200 and 300 °C to form more active oxide by decomposition and reaction with O_2_ in the air. The corresponding metal oxide, via decomposition of chelating compound, then reacted with the CaO which had formed from the decomposition of Ca(OH)_2_ during heating. This phenomenon promoted the sintering of CaO and improved the hydration resistance of CaO material.

## 2. Experimental Procedure

Active lime was used as the raw material (Wugang Refractory Company, Wuhan, China), and the chemical composition of the active lime is shown in Table 1. The pretreatment reagents were Ti chelating compound (Nanjing Capatue Chemical Company, China, CAS Number 68586-02-7) and Al chelating compound (Alfa Aesar Chemical Company, China, CAS Number 14782-75-3). The properties of the chelating compounds are shown in Table 2.

**Figure 1:**
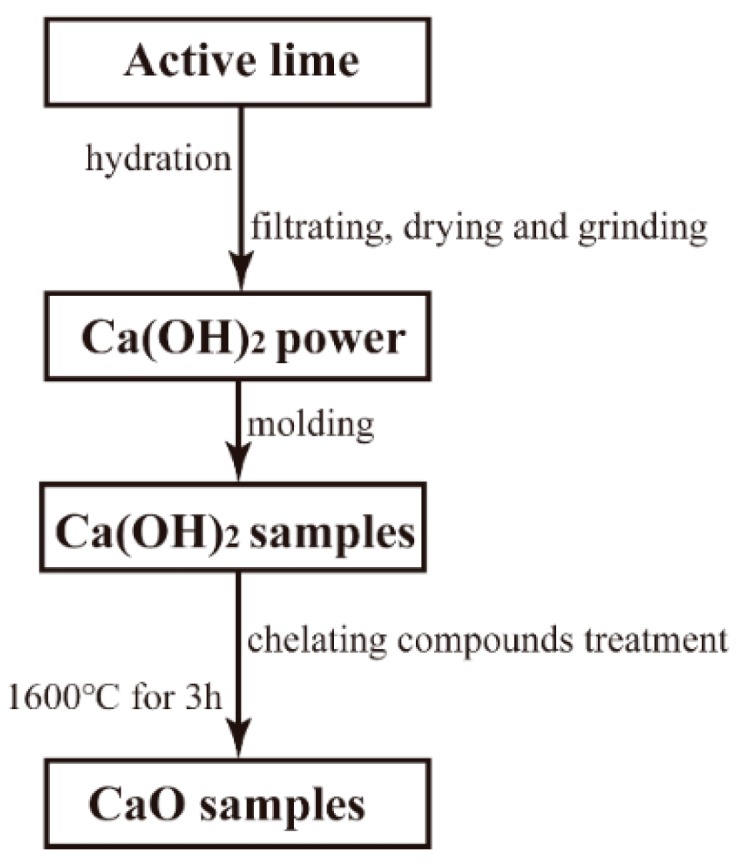
Schematic diagram of the experimental process.

Active lime was used to prepare Ca(OH)_2_ in this study. The Ca(OH)_2_ powder was prepared by hydrating, filtrating, drying, and fine grinding the active lime. The prepared Ca(OH)_2_ powder was pressed into a cylindrical sample of Ø36 mm × 20 mm under a pressure of 60 MPa. The apparent porosity of the obtained Ca(OH)_2_ sample was 43.2%. The Ca(OH)_2_ cylindrical samples were then placed into two prepared chelating compound solutions (ethanol solution of 75 mass% concentration) before being impregnated in vacuum for 10 min. After the samples were dried out, the amount of absorbed Ti chelating compound and Al chelating compound were measured to be 6.92 wt.% and 7.09 wt.%, respectively. After that, the samples were sintered at 1600 °C for 3 h. The composite structure was carried out by infiltration [25]. The experimental process is shown in Figure 1. The sample without pretreatment was named C_0_, samples pretreated with Ti and Al chelating compounds were named C_T_ and C_A_, respectively. Two measurements were used to evaluate the hydration resistance of CaO materials (the tests of CaO sample weight gain rate were done in a curing chamber and in air, respectively). Thermal analysis (TG-DSC) was used to assist the characterization of hydrated samples. Scanning electron microscopy (SEM, Nova 400 Nano-SEM, FEI Company, Hillsboro, OR, USA) and EDS spectrum analysis (INCA IE 350 Penta FET X-3, Oxford, UK) were used to assist the analysis of the sample microstructure before and after hydration test. The composition of the surface phase of CaO specimens obtained by pretreatment was studied by X-ray diffraction analysis (X’Pert Pro, Philips, Eindhoven, the Netherlands; using Ni-filtered Cu Kα radiation at a temperature of 20 °C) and X-ray photoelectron spectroscopy (Thermo Escalab 250XI, Thermo Fisher Scientific, Waltham, MA, USA). The bulk density and the apparent porosity of the specimens were measured using Archimedes’ method with kerosene.

## 3. Results and Discussion

### 3.1. Hydration Resistance of CaO Samples

**Figure 2:**
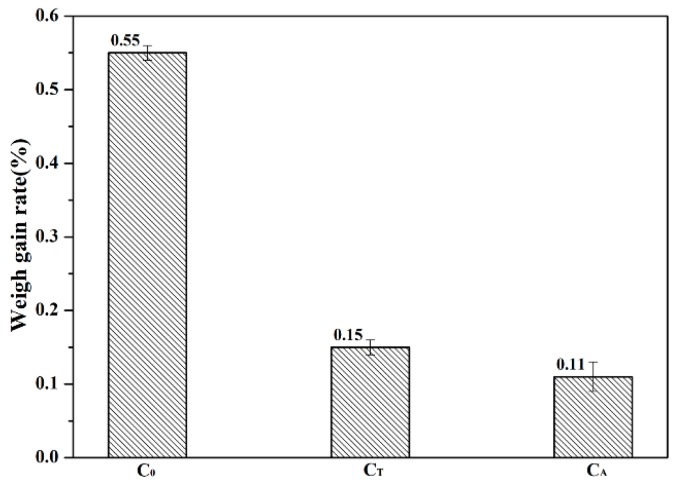
Effect of chelating compounds on the average hydration weight gain rate of CaO samples in the curing chamber.

**Figure 3:**
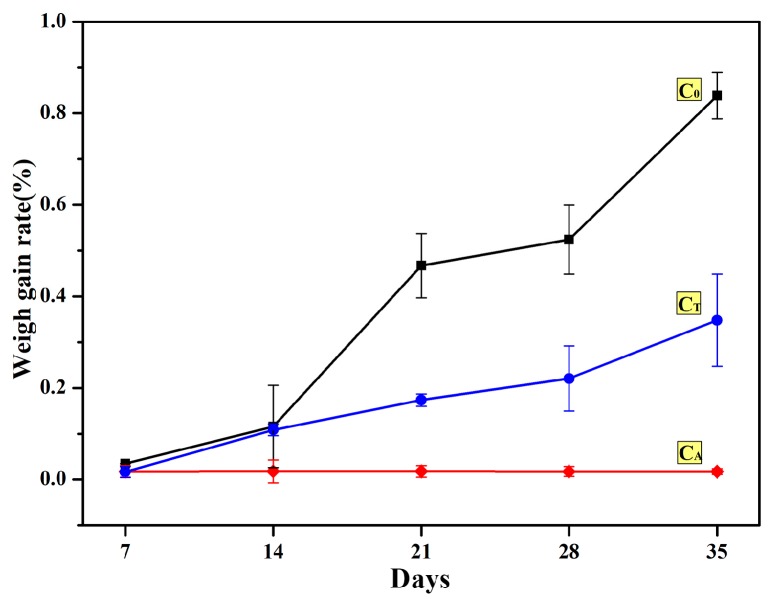
Effect of chelating compounds on the hydration weight gain rate of CaO sample in the air.

For measurement 1, specimens were placed in a chamber under a constant temperature of 50 °C and humidity of 90% for 10 h. In measurement 2, the specimens were placed in the air for 35 days. During this time, the temperature varied from 7.8 °C to 24.4 °C, and the relative humidity varied from 28% to 96%. The hydration resistance of the specimens was characterized by the weight gain rate, as in the following Equation (1):

Weight gain rate = (M_2_ − M_1_)/M_1_*100%, (1)

where M_1_ is the weight of the sample before hydration, and M_2_ is the weight of the sample after hydration.

(і) The results obtained under controlled conditions are shown in Figure 2. It could be seen from the results that the average hydration weight gain rate of sample C_0_ was 0.55%. However, the hydration weight gain rate of samples C_T_ and C_A_ decreased to 0.15% and 0.11%, respectively.

(іі) The hydration weight gain rates of the samples placed in the air are shown in Figure 3. The hydration weight gain rate of sample C_0_ increased significantly during testing and gained more than 0.8% weight in 35 days. However, the weight gain rate of sample C_T_ was about 0.3%. In particular, the weight gain rate of sample C_A_ was about 0.02% after 35 days. The results showed that both chelating compounds could significantly improve the hydration resistance of CaO samples, and the Al chelating compound was more effective.

**Figure 4:**
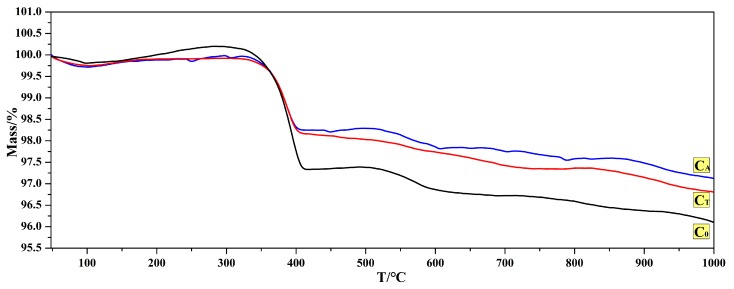
Effect of chelating compounds on the TG results of hydrated samples.

Figure 4 shows the TG results presenting mass losses of hydrated samples after being tested in measurement 1. The hydrated sample was uniformly ground and then subjected to thermal analysis under N_2_ atmosphere. The lesser mass losses in the TG result indicated the lower weight gain rates of samples C_A_ and C_T_ in the hydration test. It can be seen from Figure 4 that the mass loss of sample C_0_ was the largest and that of sample C_A_ was the smallest, meaning that the Al chelating compound enhanced the hydration resistance of CaO samples more effectively.

### 3.2. Densification

**Figure 5:**
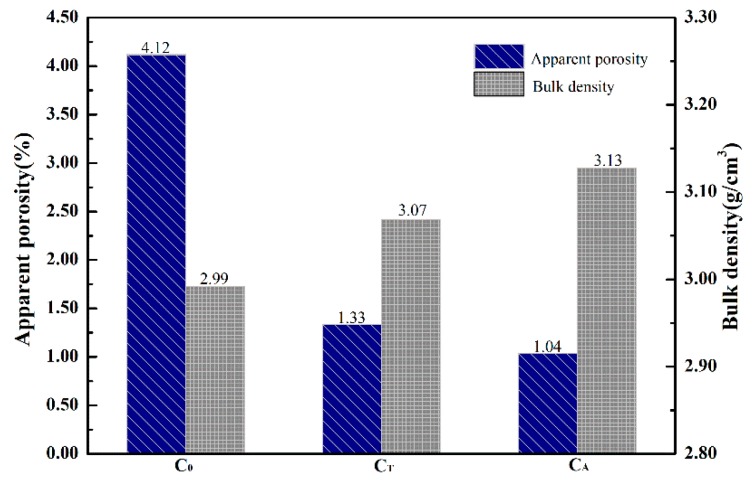
Effect of chelating compounds on the density and porosity of CaO samples

Figure 5 shows the effect of surface pretreatment on the apparent porosity and bulk density of sintered CaO samples. The bulk density of sample C_T_ increased from 2.99 g/cm^3^ to 3.07 g/cm^3^, and the apparent porosity decreased from 4.12% to 1.33% when compared with sample C_0_. At the same time, the bulk density of sample C_A_ increased to 3.13 g/cm^3^ and the apparent porosity of sample C_A_ decreased to 1.04% when compared with sample C_0_.

The densification of the samples after surface pretreatment was improved, which reduced the amounts of open pores and cracks on the surface of CaO material. Al_2_O_3_ was a decomposition product of the Al chelating compound at high temperature, which acted as a liquid-phase sintering aid to promote the grain growth of CaO, making the structure denser [26]. TiO_2_ was a decomposition product of the Ti chelating compound; it can also promote solid-phase sintering of CaO because Ti^4+^ replaces Ca^2+^ to form a solid solution at high temperature and produces Ca vacancy [VCa’’] (Equation (2)) [27]. The diffusion and migration of atoms are the basic factors for sintering. The generation of vacancies provides a diffusion source of particles. This can improve the self-diffusion coefficient and the vacancy diffusion coefficient. This is beneficial to the diffusion and migration of the particles, and promotes sintering [28].

(2) $${\mathrm{TiO}}_{2}\overset{\mathrm{CaO}}{\to }{\mathrm{Ti}}^{▪▪}{}_{\mathrm{Ca}}{+V\prime \prime }_{\mathrm{Ca}}{+2O}_{O}$$

### 3.3. Phase Analysis of CaO Samples

**Figure 6:**
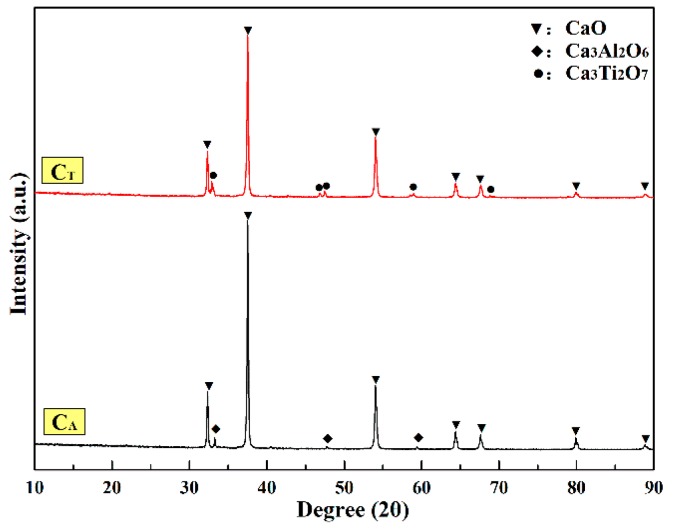
XRD phase analysis of CaO surface pretreated by chelating compounds.

**Figure 7:**
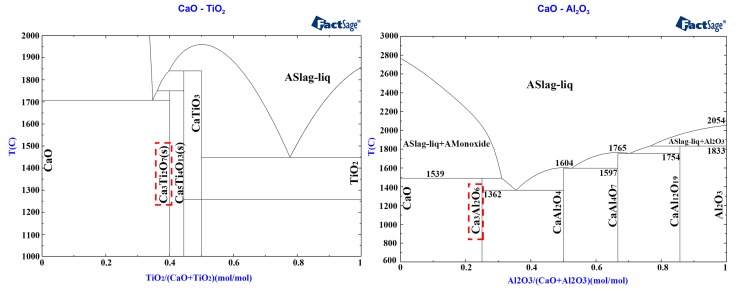
Phase diagrams of CaO-TiO_2_ and CaO-Al_2_O_3._

Figure 6 shows the XRD patterns on the surface of the samples sintered at 1600 °C for 3 h. The figure indicates that Ca_3_Ti_2_O_7_ was formed on the surface of the sample C_T_, and it promoted the sintering density of the CaO sample. A small amount of Ca_3_Al_2_O_6_ was formed on the surface of the sample C_A_, which had a positive effect on preventing the surface of the CaO sample from contacting with water vapor. Ca_3_Ti_2_O_7_ and Ca_3_Al_2_O_6_ phases were found in the CaO-TiO_2_ and CaO-Al_2_O_3_ phase diagrams (Figure 7). The surface treatment of CaO material slowed down the hydration process in two aspects. The first was in the formation of a small amount of water-resistant compounds on the surface of CaO which filled the pores on the surface, reducing the contact area between CaO grains and water vapor. The second was in the promotion of the sintering degree of the surface portion of the CaO sample, which reduced the porosity and the probability of defect formation.

**Figure 8:**
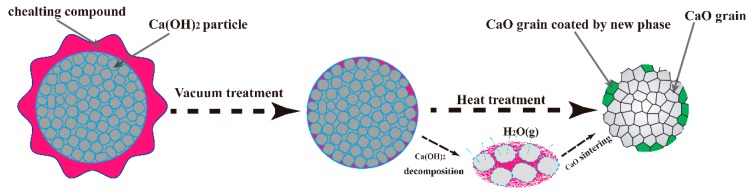
Mechanism of effects of chelating compound on hydration resistance of CaO material.

Figure 8 shows the mechanism of the effects of chelating compounds on the hydration resistance of CaO. The samples were placed in the chelating compound solution under vacuum conditions. The chelating compound penetrated into the open pores of the Ca(OH)_2_ sample and coated the surface of the sample; it was then pyrolyzed into an oxide and reacted with CaO to form a new phase, which promoted the sintering of the sample.

**Figure 9:**
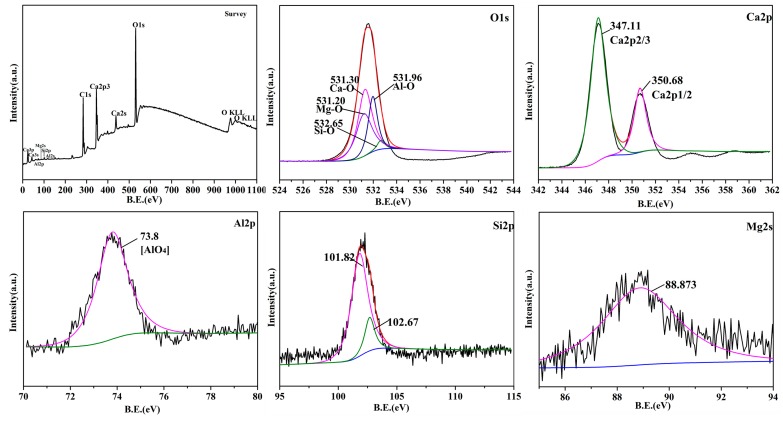
X-ray photoelectron spectroscopy (XPS) analysis on the CaO surface of sample C_A_.

Figure 9 shows the X-ray photoelectron spectroscopy (XPS) analysis on the surface of CaO sample C_A_ (C1s was the introduced carbon correction peak). From the chemical shift in the binding energy of O 1p, Ca 2p, Al 2p, Si 2p, and Mg 2s, the oxygen was present in the form of M-O (M included Ca, Al, Si, and Mg). In particular, the existence of Al was found mostly in the form of [AlO_4_] tetrahedron (73.8 eV) [29] (possibly containing a small amount of [AlO_6_] octahedron). Compared with the tetrahedral [AlO_4_] and octahedral [AlO_6_] mixed structure of Ca_3_Al_2_O_6_, the hole in the lattice [AlO_4_] was smaller. That might make it more difficult for OH^-^ to enter the crystal interior and slowed down the sample’s hydration rate. The improvement of the hydration resistance of sample C_A_ might also be attributed to the substitution of Si-O (101.82 eV) or Mg-O (88.873 eV) for the part of Al-O inside of the Ca_3_Al_2_O_6_ crystal that existed on the surface of CaO sample.

### 3.4. Microstructural Analysis of CaO Material

**Figure 10:**
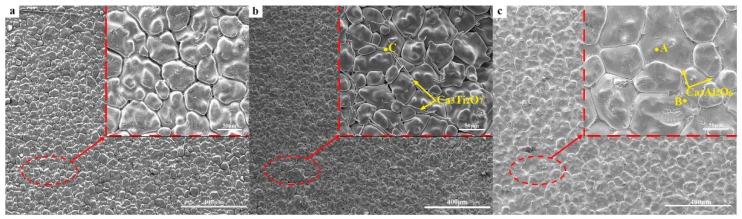
SEM images of CaO samples: (**a**) C_0_; (**b**) C_T_; (**c**) C_A_.

Figure 10 shows images of the samples’ surface microstructure after having been heated at 1600 °C for 3 h. Table 3 shows the energy-dispersive spectrometry (EDS) results of points A, B, and C in Figure 10. The average gap sizes of C_0_, C_T_, and C_A_ were respectively 4.62, 2.31, and 1.44 μm. It can be seen that the gaps between the CaO grain boundaries in sample C_T_ and C_A_ were smaller than those in sample C_0_. Sample C_A_ had the most compact structure. Ca_3_Ti_2_O_7_ was formed at the grain surface and the grain boundary, filling pores and enhancing the degree of crystal bonding. For sample C_A_, the surface grain size development was significantly greater than that of sample C_0_, which increased the grain size of CaO from 50 to 70–100 μm. Ca_3_Al_2_O_6_ was formed on the grain boundary and filled triple points, which increased the density of the crystal. The formation of a liquid phase at high temperatures facilitated grain growth because of the faster migration rate of atoms in the liquid phase. Although Ca_3_Al_2_O_6_ was also hydratable, it converted some free CaO to Ca_3_Al_2_O_6_, and the hydration resistance was better than that of CaO-covered defects sites [30]. Chelating compounds promoted the bonding of CaO grains on the surface and formed a protective coating to reduce the contact area between CaO and water vapor, thus enhancing the hydration resistance of the CaO sample.

**Figure 11:**
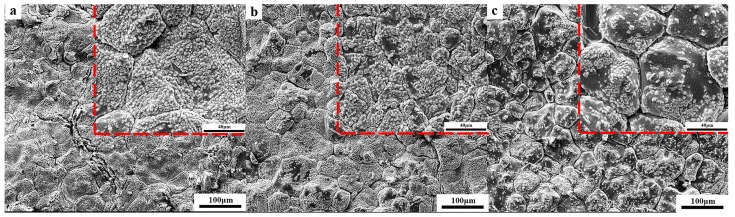
SEM images of sample surfaces placed in the air for 7 days: (**a**) C_0_; (**b**) C_T_; (**c**) C_A_.

Figure 11 shows the surface microstructure of the samples after being placed in air for 7 days. The dark parts in Figure 11 are unhydrated CaO particles, and the light parts covering the black CaO grains are Ca(OH)_2_ particles. Figure 11a shows the hydration of the sample C_0_, and there were even cracks due to hydration. It can be seen from Figure 11c that the CaO in the C_A_ sample showed little hydration. This also confirmed that the hydration resistance of the CaO sample pretreated by the Al chelating compound was the best of all the tested samples.

**Figure 12:**
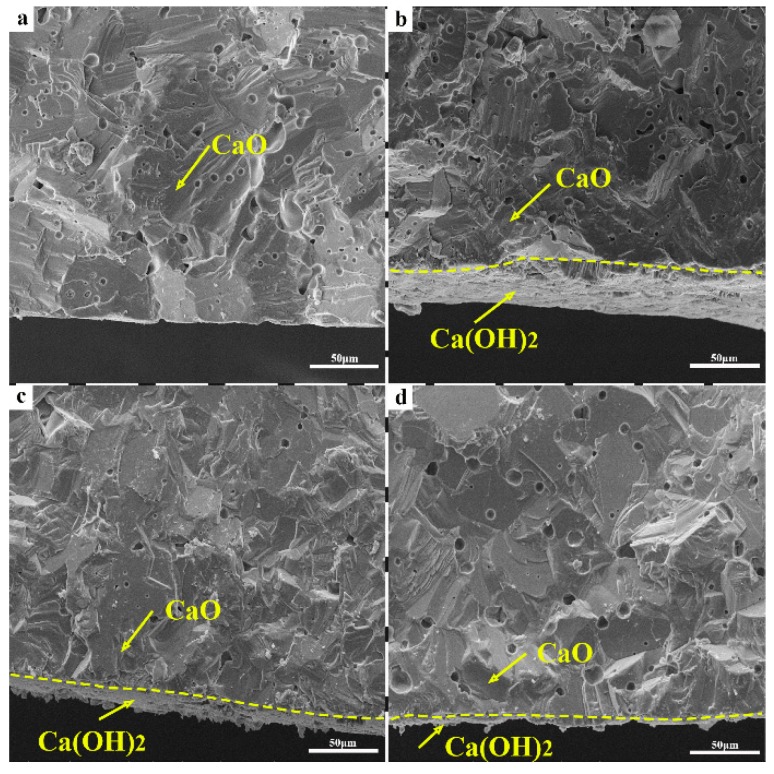
SEM photo of the sample section placed in the air before and after 14 days (**a**) Sample C_0_ before hydration test; (**b**) Sample C_0_ after hydration test; (**c**) Sample C_T_ placed in the air after 14 days; (**d**) Sample C_A_ placed in the air after 14 days.

Figure 12 shows cross-sectional SEM images of CaO samples before and after the hydration test. It shows that Ca(OH)_2_ layers were formed on the surface of the CaO samples after the hydration. The Ca(OH)_2_ layer formed in Figure 12d (sample C_A_) was the thinnest, indicating the lowest reaction degree of CaO and water vapor. Therefore, the hydration resistance of the CaO sample pretreated by Al chelating compound was greatly improved.

## 4. Conclusions

Both Ti and Al chelating compounds enhanced the hydration resistance of the CaO material significantly. At the same time, both promoted the surface sintering of CaO and reduced the contact area of CaO with water vapor, making the surface structure much denser. Chelating compounds decomposed at high temperature, and the decomposition products reacted with CaO to form water-resistant compounds which filled the pores and cracks located on the surface of the CaO material. Comparing the experimental results, it can be stated that the pretreatment with Al chelating compound on the surface of CaO sample improved the hydration resistance of CaO material tremendously.

## Figures and Tables

**Table 1:** Chemical composition of active lime (wt.%).

Raw Material	CaO	SiO_2_	Al_2_O_3_	Fe_2_O_3_	MgO	K_2_O	Na_2_O	TiO_2_	L.O.I.
Active lime	96.80	0.44	0.47	0.30	1.59	0.007	0.007	0.005	0.23

**Table 2:** Physicochemical properties of chelating compounds.

Item	Molecular Formula	Ti (Al) Content (%)	Relative Molecular Mass	Density (g/cm^3^)
Ti chelating compound	C_15_H_26_TiO_6_	9.8	274.29	1.040
Al chelating compound	C_12_H_23_AlO_5_	8.9	350.25	0.975

**Table 3:** EDS analyses of points A, B, and C in Figure 10.

Element	Point A (wt.%)	Point B (wt.%)	Point C (wt.%)
O	45.07	54.06	66.79
Ca	54.81	28.54	21.09
Al	0.12	17.40	-
Ti	-	-	12.12
